# Constitutive Phosphorylation of Interferon Receptor A-Associated Signaling Proteins in Systemic Lupus Erythematosus

**DOI:** 10.1371/journal.pone.0041414

**Published:** 2012-07-30

**Authors:** Gabriela Ramírez-Vélez, Francisco Medina, Luis Ramírez-Montaño, Abraham Zarazúa-Lozada, Ramiro Hernández, Luis Llorente, José Moreno

**Affiliations:** 1 Research Unit on Autoimmune Diseases, Hospital de Especialidades, Centro Médico Nacional Siglo XXI, Instituto Mexicano del Seguro Social (IMSS), Mexico, D. F., Mexico; 2 Facultad Mexicana de Medicina, Universidad LaSalle, México, D. F., Mexico; 3 Research Unit on Pharmacology Hospital de Especialidades, Centro Médico Nacional Siglo XXI, Instituto Mexicano del Seguro Social (IMSS), Mexico, D. F., Mexico; 4 Department of Rheumatology, Hospital de Especialidades, Centro Médico Nacional Siglo XXI, Instituto Mexicano del Seguro Social (IMSS), Mexico, D. F., Mexico; 5 Department of Immunology and Rheumatology, Instituto Nacional de Ciencias Médicas y Nutrición Salvador Zubirán, Mexico, D. F., Mexico; Oklahoma Medical Research Foundation, United States of America

## Abstract

**Background:**

Overexpression of type I interferon (IFN-I)-induced genes is a common feature of systemic lupus erythematosus (SLE) and its experimental models, but the participation of endogenous overproduction of IFN-I on it is not clear. To explore the possibility that abnormally increased IFN-I receptor (IFNAR) signaling could participate in IFN-I-induced gene overexpression of SLE, we examined the phosphorylation status of the IFNAR-associated signaling partners Jak1 and STAT2, and its relation with expression of its physiologic inhibitor SOCS1 and with plasma levels of IFNα and IFN-like activity.

**Methodology/Principal Findings:**

Peripheral blood mononuclear cells (PBMC) from SLE patients with or without disease activity and healthy controls cultured in the presence or in the absence of IFNβ were examined by immunoprecipitation and/or western blotting for expression of the two IFNAR chains, Jak1, Tyk2, and STAT2 and their phosphorylated forms. In SLE but not in healthy control PBMC, Jak1 and STAT2 were constitutively phosphorylated, even in the absence of disease activity (basal pJak1: controls vs. active SLE p<0.0001 and controls vs. inactive SLE p = 0.0006; basal pSTAT2: controls vs. active and inactive SLE p<0.0001). Although SOCS1 protein was slightly but significantly decreased in SLE in the absence or in the presence of IFNβ (p = 0.0096 to p<0.0001), in SOCS1 mRNA levels were markedly decreased (p = 0.036 to p<0.0001). IFNβ induced higher levels of the IFN-I-dependent MxA protein mRNA in SLE than in healthy controls, whereas the opposite was observed for SOCS1. Although there was no relation to increased serum IFNα, active SLE plasma could induce expression of IFN-dependent genes by normal PBMC.

**Conclusions/Significance:**

These findings suggest that in some SLE patients IFN-I dependent gene expression could be the result of a low IFNAR signaling threshold.

## Introduction

Systemic lupus erythematosus (SLE) is a chronic multiorgan autoimmune disease with multiple defects of the immune system [1], [2]. Genetic studies of large SLE cohorts have shown association to polymorphisms of genes coding for proteins with great functional diversity [3], [4], [5], [6], [7] with nearly 30 described to date [8]. Gene associations for distinct SLE patients are highly variable, which could explain its complex pathogenesis and clinical heterogeneity. Nevertheless, a common finding in SLE and its mouse models is activation of type I interferon (IFN-I) dependent pathways. SLE patients can have increased serum IFNα levels, especially during disease activity [9], [10], [11], [12]. Gene expression microarray studies revealed overexpression of IFN-inducible genes (IFN-signature) in SLE [13], [14], [15]. Moreover, some SLE-associated polymorphisms occur in genes related to the IFN-I pathway [6], [16], [17].

Therapeutic use of IFN-I for non-autoimmune disorders can induce autoantibodies typical of SLE (∼22%), autoimmunity (∼19%) or overt SLE (∼0.7%) [18], [19]. However, increased IFN-I by itself does not explain SLE, as in most people exogenous IFN-I or its production in response to viral infection does not lead to SLE and IFN-I-induced SLE is uncommon. Therefore, in some SLE patients, additional susceptibility traits could lead to an intrinsically enhanced response to IFN. In humans, IFN-I are encoded by ∼15 IFNα genes, and one each IFNβ, ω and κ [20], which exert their biological effects through an ubiquitously expressed IFNAR formed by the IFNAR1 and IFNAR2 chains. The canonical IFNAR signaling pathway involves activation of the Janus kinases (Jak) Tyk2 and Jak1 to the cytoplasmic tails of IFNAR1 and IFNAR2 respectively, creating binding sites for signal transduction and activators of transcription (STAT) 1 and 2 to yield STAT1-2 heterodimers and STAT1 homodimers [21], which translocate to the cell nucleus [22], [23] to form, together with IRF9, the complex that drive the transcription of IFN-I responsive genes [21], [22], [23], [24]. The IFN signature comprises several chemokines, cytokines, plus additional molecules, including transcription factors and antiviral proteins, among others [25], [26].

Jak-STAT signal-transduction is negatively regulated, among others, by suppressor of cytokine signaling (SOCS) proteins (SOCS1 to SOCS7 and CIS [27], [28], [29], [30]), which down-regulate Jak-STAT by means of an U3 ubiquitin ligase domain and by a Jak inhibitory domain (SOCS1 and SOCS3). Transcription of SOCS genes occurs in a classic cytokine-Jak-STAT-induced negative feedback loop [31]. SOCS1-KO mice die neonatally with peripheral T cell activation and generalized T cell infiltrates [32], whereas partial SOCS1 deficiency in lymphoid cells is not lethal, but leads to increased sensitivity to cytokine signaling and a SLE-like autoimmune phenotype [32], [33], [34]. IFNAR signaling is mainly regulated by SOCS1 [35], [36].

As SLE is characterized by what appears to be an increased sensitivity to otherwise normal levels of cytokines, we examined the basal phosphorylation levels of the IFNAR signaling pathway and its relationship with expression of its inhibitor SOCS1 in SLE patients. We found that SLE patients display constitutive phosphorylation of Jak1 and STAT2 compared to healthy controls, regardless of disease activity. We also found decreased SOCS1 protein and mRNA expression in SLE, but with not clear relationship with IFNAR phosphorylation.

## Results

### Constitutive Phosphorylation of Jak1 and STAT2 in SLE

**Figure 1:**
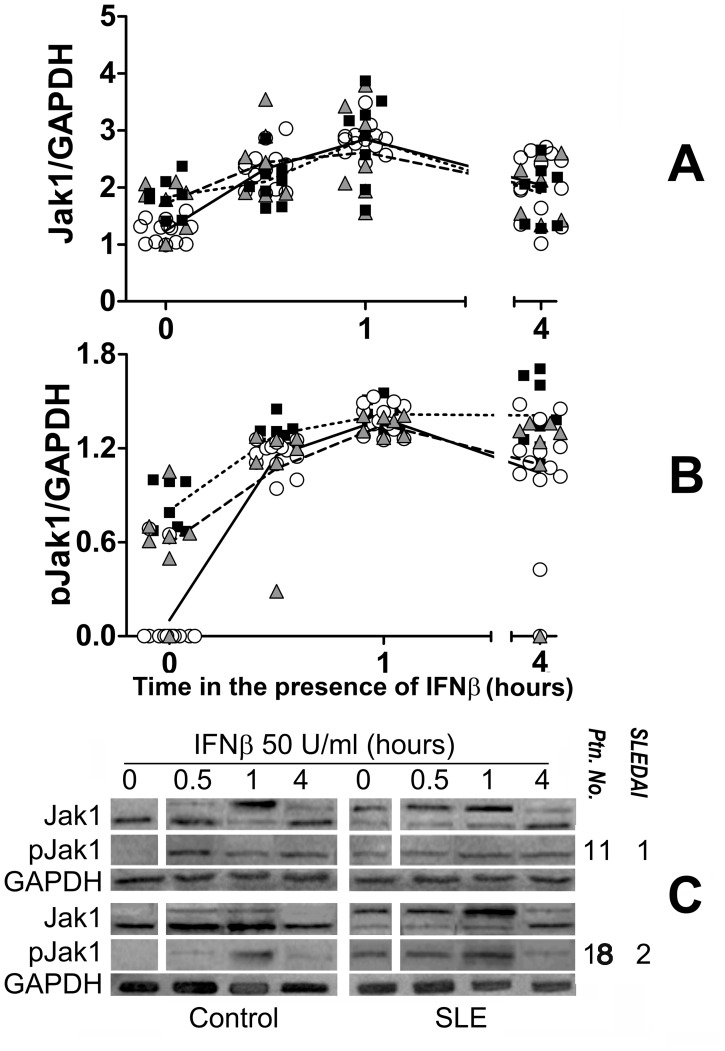
Constitutive phosphorylation of Jak1 in SLE. A and B, densitometric values of: Jak1/GAPDH and pJak1/GAPDH ratios, respectively, of PBMC protein extracts from SLE patients (n = 15, closed squares and triangles) or the same number of healthy controls (open circles) before (time 0) and at different lengths of culture after the addition of 50 U/ml human recombinant IFNβ. Inactive SLE (triangles) includes patients with SLEDAI 0 and active SLE (squares) comprises patients with SLEDAI≥1. C. Jak1 western blot samples of two patients (right) with their respective activity scores (SLEDAI) and healthy controls (left).

**Figure 2:**
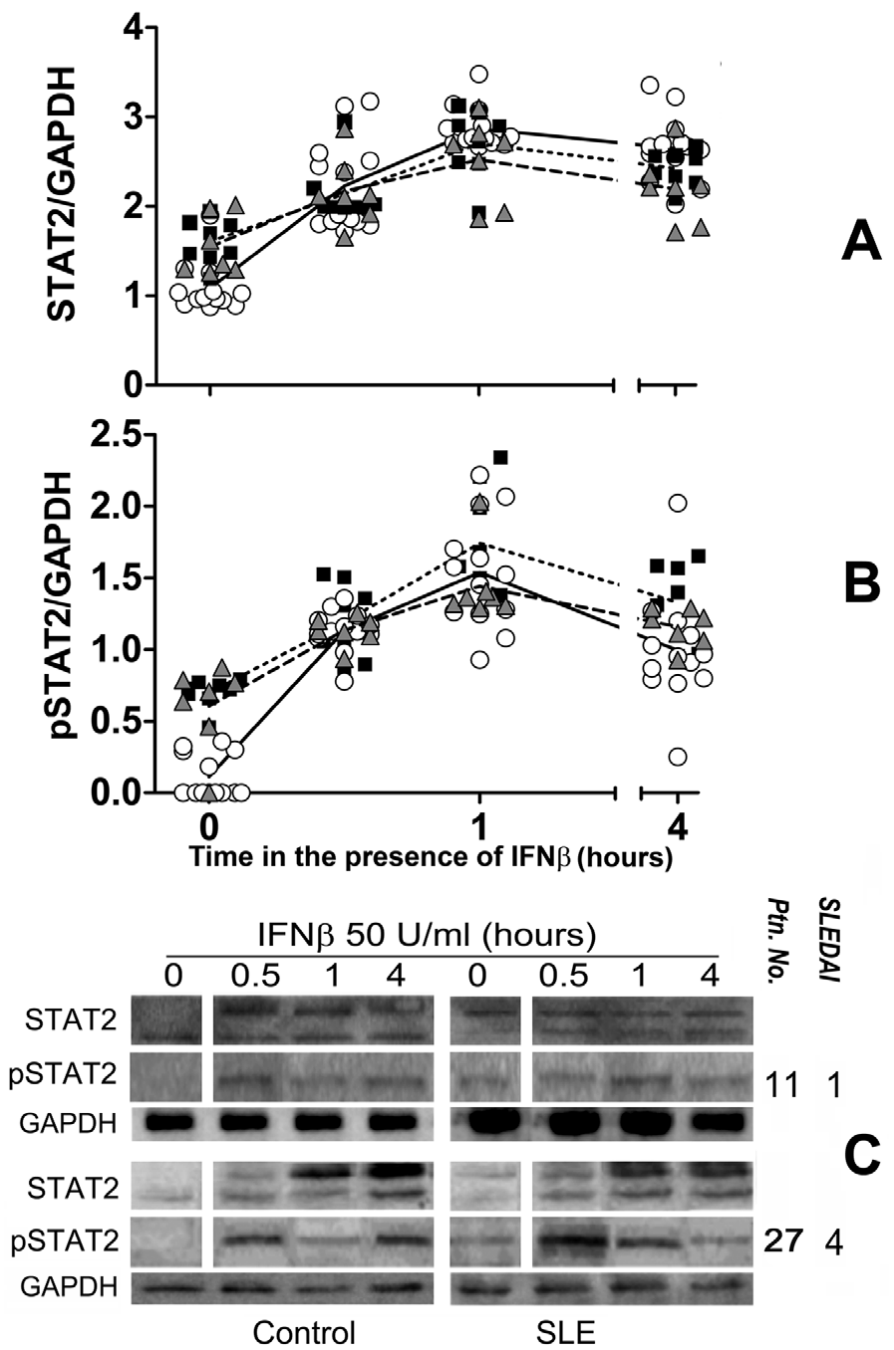
Constitutive phosphorylation of STAT2 in SLE. A and B, densitometric values of: STAT2/GAPDH and pSTAT2/GAPDH ratios, respectively, of PBMC protein extracts from SLE patients (n = 15, closed squares and triangles) or the same number of healthy controls (open circles) before (time 0) and at different lengths of culture after the addition of 50 U/ml human recombinant IFNβ. Inactive SLE (triangles) includes patients with SLEDAI 0 and active SLE (squares) comprises patients with SLEDAI≥1. C. STAT2 western blot samples of 2 patients (right) with their respective activity scores (SLEDAI) and healthy controls (left).

We first examined the status of the IFNAR associated signal transduction molecules in SLE patients. As the IFNAR1-associated signaling molecules (STAT1 and Tyk2) are also activated via the IFNγ receptor and patient’s blood supply was limiting, we assumed that examining the IFNAR2-associated proteins Jak1-STAT2 would faithfully reflect IFN-I-induced signaling. Figs. 1A, 1C and 2A, 2C, show the results of WB analysis with anti-Jak1 and STAT2 antisera of SLE or healthy control PBMC cell lysates. Basal levels of Jak1 and STAT2 were slightly but significantly higher in SLE patients. As several of the Jak1 and STAT2 WB turned out with two bands, for these studies both bands were used for densitometric quantification. Therefore, values given in Figs. 1 and 2 correspond to the sum of both bands. We assumed that the upper band corresponds to the phosphorylated forms of Jak1 and STAT2 as described [37], which increases in intensity after culture with IFNβ. This is also suggested by its location related to the molecular weight markers (not shown) run at the same time on SDS-PAGE (Precision Plus Protein Dual Colors Standards, product No. 161-0374, Bio-Rad Laboratories, Hercules, CA). However, to examine more precisely the relative abundance of each phosphorylated protein, we performed a separate analysis of phospho-Jak1 (pJak1) and phopsho-STAT2 (pSTAT2) in membranes incubated with specific antibodies against pJak1 and pSTAT2.

IFNAR in cells from SLE patients was constitutively activated *in vivo* because in un-stimulated SLE but not in healthy PBMC Jak1 was phosphorylated (p<0.0001 for all SLE patients Figs. 1B and 1C, Tables S1 and S4). This was not due to differential expression of IFNAR as both chains were similarly expressed in SLE patients and healthy controls (Fig. S1). Importantly, Jak1 was phosphorylated in inactive SLE patients (controls vs. active SLE p<0.0001 and controls vs. inactive SLE p = 0.0006) with differences between active and inactive SLE being statistically less significant (p = 0.0477). Similar results were obtained for STAT2 (Figs. 2B and 2C, Tables S2 and S5). In SLE PBMC, basal STAT2 phosphorylation was 3 to 4 times higher than in control PBMC (p<0.0001), and again both active and inactive SLE were significantly different from healthy controls (p<0.0001). STAT2 phosphorylation differences between active and inactive SLE patients were not significant and there was no correlation between SLE disease activity and Jak1 or STAT2 phosphorylation (Figs. S2 and S3).

### IFN-I-like Activity in SLE Plasma and its Association with Disease Activity but not with Jak1 Phosphorylation

**Figure 3:**
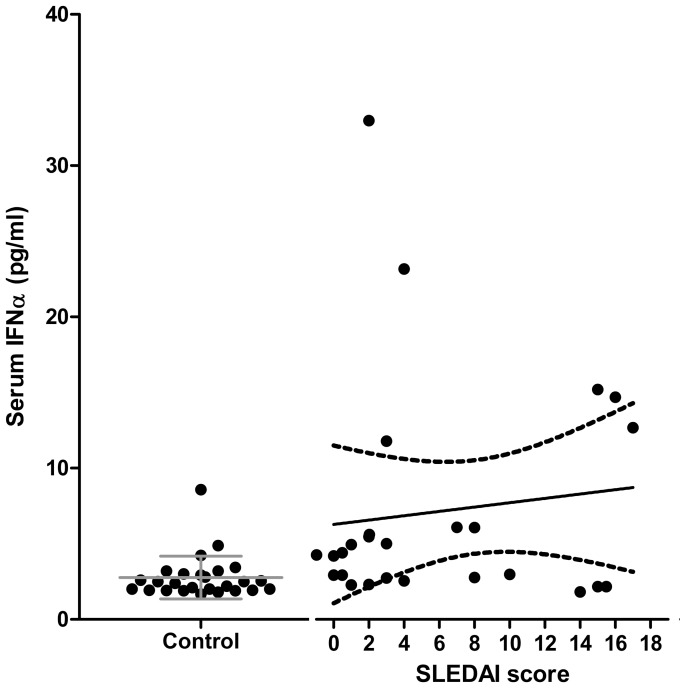
Serum IFNα is not uniformly increased in SLE patients and does not correlate with disease activity. Serum samples from 26 SLE patients (right) and an equal number of age and sex-matched healthy controls (left) were used to determine levels of IFNα with a commercial kit that detects 14 of the 15 known isoforms of the cytokine. Data from SLE patients were plotted to examine the correlation with disease activity according to the SLEDAI scores and are expressed as picograms/milliliter. R^2^ = 0.03641.

**Figure 4:**
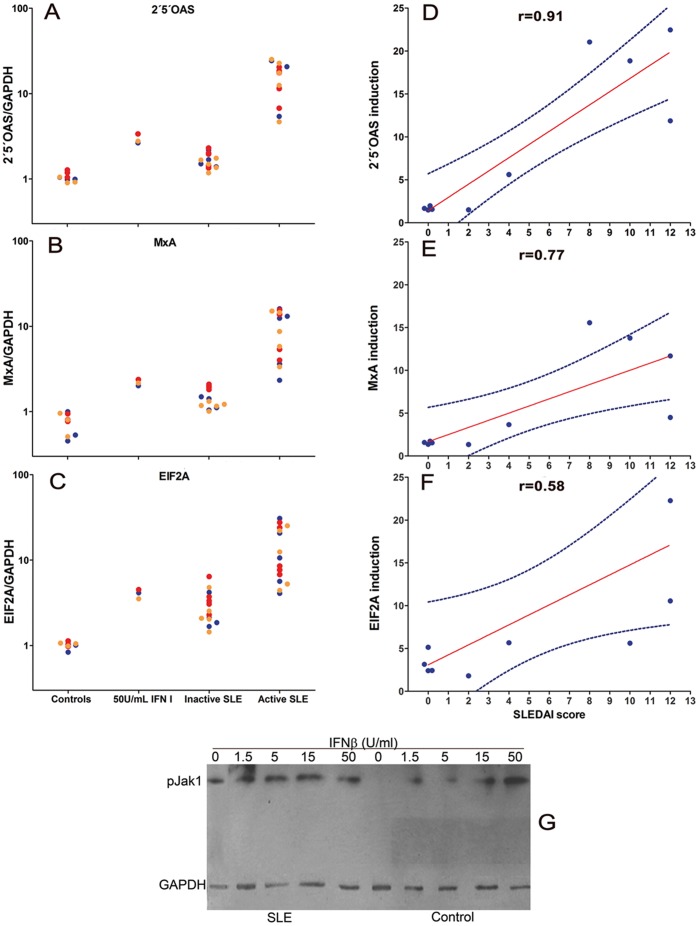
Active SLE plasma induce the expression of IFN-I dependent genes. PBMC from three healthy donors were individually stimulated for 1 h with plasma from obtained from 5 inactive SLE, 5 active SLE patients, 3 healthy controls or 50 U/ml recombinant IFNαβ. Induction of the IFN-I dependent genes 2′5′OAS (**A**), MxA (**B**) or EIF2α (**C**) was examined by qRT-PCR (SYBR-Green). Graphs show the induction on PBMC from three healthy donors (red, yellow and blue circles indicate different donors) for the indicated gene, under the indicated conditions for each column (control plasma, 50 U/ml IFNββ and plasma from inactive or active SLE). Data are expressed as the x-fold induction of each mRNA compared to unstimulated PBMC. **D**, **E** and **F**: Pearson’s correlation between induction of each gene (mean of PBMC from three healthy donors) and SLEDAI scores. **D** = 2′5′OAS, **E** = MxA, **F** = EIF2α. **G**: Western blot analysis of one SLE patient (left) and one healthy control (right)-PBMC lysates cultured for 1 hour with increasing concentrations of IFNβ (0, 1.5, 5, 15 and 50 U/ml, blotted with anti-pJak1 antibody.

Constitutive phosphorylation of Jak1 and STAT2 could be due to the presence of IFN in the serum of SLE patients, which seemed unlikely for inactive SLE as it has been shown that increased IFN-I in SLE is associated to disease activity. Fig. 3 shows that although some active SLE patients had increased levels of IFNα, these did not correlate with the SLEDAI scores, and in no case an inactive SLE patient had IFNα levels above those of control PBMC. Nevertheless, it has been found that active SLE plasma contains IFN-I-like activity that can be neutralized with anti-IFNα blocking antibodies [38]. Figure 4A, B and C shows indeed that active SLE plasma induces high mRNA expression of three IFN-I-dependent genes, namely 2′5′-oligo-A synthetase (2′5OAS), myxovirus resistance A (MxA) and eukaryotic initiation factor (EIF2α). However, only a few inactive SLE plasmas could induce these genes but to a much lesser degree. Moreover, induction of these genes, particularly 2′5OAS, by plasma correlated with SLEDAI scores (Fig. 4D, E and F). Thus, although in some inactive SLE Jak1 and STAT2 phosphorylation could be due to IFN-I-like activity, some inactive SLE plasmas did not induce expression of these IFN-I-responsive genes by PBMC from three different healthy donors, suggesting that, at least in some SLE patients, Jak1 and STAT2 phosphorylation is constitutive and independent of exogenous IFN. Indeed, patient 21 (Table 1), who had a SLEDAI score of 2, lacked plasma IFNα as well as 2′5OAS, MxA or EIF2α-inducing activity showed marked phosphorylation of Jak1 in the absence of exogenous IFN-I, which no further increased with the addition of 50 U/ml IFNβ (Fig. 4G).

**Table 1:** SLE Patients Included in This Study.

Ptn. No.	Age	SLEDAI	Years/SLE		C3	C4	PDNP^1^	Other
1	33	0	10	Normal		Normal	0	Nil
2	41	0	23	Normal		Normal	0	Nil
3	32	0	3	Low		Low	0	Nil
4	30	0	15	Normal		Normal	0	Nil
5	32	0	9	Normal		Low	0	Nil
6	23	0	7	Normal		Low	0	Nil
7	28	0	0.3	Normal		Normal	0	Nil
8	42	0	1	Normal		Normal	5	Nil
9	27	0	12	Low		Normal	5	Nil
10	22	0	7	ND		ND	5	HCQ 150
11	50	0	14	ND		ND	0	Nil
12	43	0	1	Normal		Normal		AZA 50
13	30	0	12	Normal		Normal	2.5	Nil
14	46	0	14	Normal		Normal	5	Nil
15	48	1	1	Normal		Normal	5	Nil
16	36	1	10	Low		Low	7.5	Nil
17	36	1	15	Normal		Normal	10	Nil
18	20	2	2	Normal		Normal	2.5	Nil
19	21	2	5	Normal		Low	5	Nil
20	20	2	7	Low		Low	10	Nil
21	19	2	6	Low		Normal	5	Nil
22	15	3	1	Low		Low	5	Nil
23	22	3	4	Normal		Low	7.5	HCQ 150
24	24	3	3	Low		Low	0	HCQ 200
25	23	3	11	Low		Low	7.5	HCQ 150
26	19	3	1	Normal		Low	7.5	HCQ 150
27	28	4	1	Low		Low	5	Nil
28	40	4	0.6	Low		Low	7.5	HCQ 400
29	42	4	8	Low		Normal	7.5	Nil
30	49	7	6	Low		Normal	0	AZA 50; HCQ 300
31	44♂	8	5	Low		Normal	5	Nil
32	35	8	1	Low		Normal	0	HCQ 300
33	30	8	16	Low		Low	7.5	AZA 75
34	20	10	3	Low		Low	2.5	CHQ 100
35	36	10	15	Low		Low	10	AZA 75
36	41	12	2	Low		Low	10	AZA 50
37	33	12	16	Low		Low	15	Nil
38	34	14	7	Low		Low	10	CHQ 200
39	25	15	15	Low		Low	5	CHQ 200
40	50	15	3	Low		Low	7.5	Nil
41	35	15	2	Low		Normal	50	CHQ 200
42	20	16	<1	Low		Low	7.5	CHQ 200
43	17	17	<1	Low		Low	0	Nil

1 mg per day of prednisone. HCQ = hidroxichloroquine; AZA = Azatioprine; CHQ = chloroquine.

Constitutive phosphorylation of Jak1 and STAT2 in SLE PBMC suggested two non-exclusive interpretations: either an intrinsically increased Jak1 kinase activity or decreased regulation. Therefore, Jak1 and STAT2 phosphorylation was examined by culturing PBMC with 50 U/ml IFNβ (Figs. 1B and C, 2G, and 3B and C). As seen, IFNβ induced Jak1 and STAT2 phosphorylation in both healthy control and SLE PBMC. In SLE cells, increase phosphorylation was only marginal, whereas in healthy controls, one hour after the addition of IFNβ their Jak1 and STAT2 phosphorylation reached similar levels to those of SLE PBMC. Interestingly, 4 hours after the addition of IFNβ, Jak1 and STAT2 remained phosphorylated in SLE but not in control cells. Thus, phosphorylation of Jak1 and STAT2 in SLE patients does not appear to be due to increased proximal IFNAR signaling potential but rather to impaired receptor regulation. These findings suggest that impaired IFNAR regulation could be a SLE susceptibility trait.

### Decreased Expression of SOCS1 in SLE

**Figure 5:**
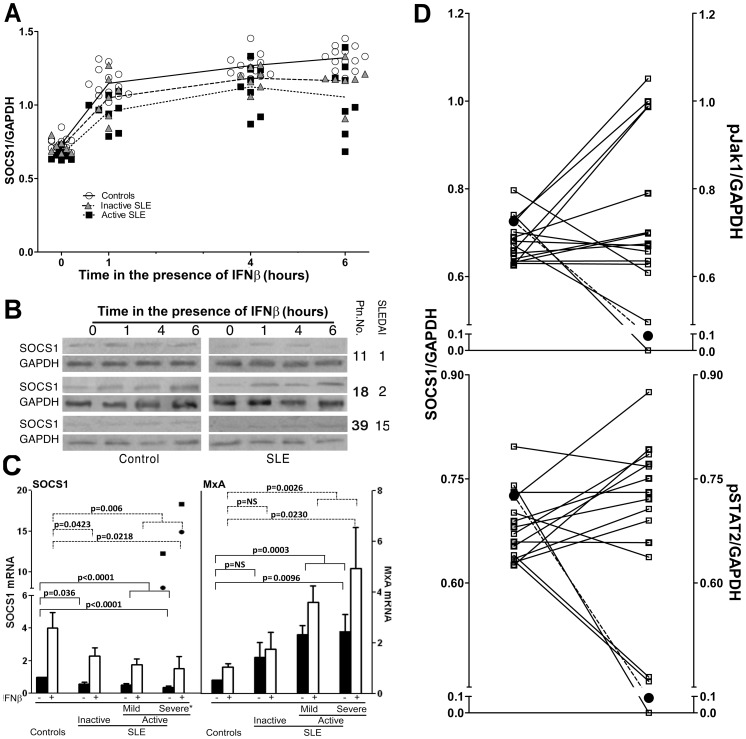
Decreased expression of SOCS1 protein and mRNA in SLE and its relation to Jak1 and STAT2 phosphorylation. A. Densitometic values of SOCS1 western blots in SLE or healthy controls (open circles) before (time 0) and at different lengths of culture after the addition of 50 U/ml human recombinant IFNββ. Triangles = inactive SLE (SLEDAI 0), squares = active SLE (SLEDAI≥1). B. Representative Western blot samples of 6 patients (right) with their respective activity scores (SLEDAI) and healthy controls (left). C. Real time RT-PCR achieved with total RNA from SLE (n = 31) or an equal number of healthy control PBMC before (black bars) or after (white bars) 30 min of culture in the presence of 50 U/ml IFNβ. Expression of SOCS1 is depicted on the left panel and MxA on the right. Inactive SLE = SLEDAI 0 (n = 9), active mild =  SLEDAI 1–4 (n = 12) and active severe = SLEDAI>4 (n = 10). Statistical values were obtained by Student’s t test for non-paired samples. The two points shown in the SOCS1 bars above the severely active patients correspond to the only ones with increased SOCS1 expression and are not included within the corresponding bars and were not included for statistics (see text). D. Individual comparisons of basal levels of SOCS1 (left) to pJak1 (top) and pSTAT (bottom) of individual SLE patients (n = 15), represented as open squares. The mean values of healthy controls (n = 15) are represented as black circles.

The finding of constitutive Jak1 and STAT2 phosphorylation in SLE led us to examine further this phenomenon. We chose to examine SOCS1 because it is the best characterized inhibitor of cytokine receptors, including IFNAR, and because its partial deficiency leads to a SLE-like syndrome in experimental models [32], [33], [34], [39]. Figure 5A shows results of western blot analysis with anti-SOCS1 antiserum of SLE or control PBMC protein extracts. Although SOCS1 was decreased in all SLE patients, only differences between active SLE patients and healthy controls were significant (p = 0.0007). As SOCS1 expression can be induced by IFN-I, we examined SOCS1 expression in the presence of IFNβ. Fig. 5A shows that IFNβ induced lower levels of SOCS1 in SLE PBMC than in healthy controls (after 6 h in the presence of IFNβ, controls vs. active SLE p<0.0001; controls vs. inactive SLE p = 0.0008; see Tables S3 and S6 for all values and statistical data), which, again, was not related to disease activity (Fig. 5B shows three representative blots). Culture in the absence of IFN-I failed to induce SOCS1 in control PBMC (data not shown). There was no correlation between decreased SOCS1 protein expression by SLE PBMC and disease activity (SLEDAI scores), either in the absence or in the presence of IFNβ (Fig. S4). Taken together, these results indicate that in SLE patients, basal levels of SOCS1 protein expression and its induction by IFNβ are decreased.

However, in spite the statistical significance of decreased SOCS1 protein in SLE, the differences observed by western blot analysis seemed rather small. Therefore, we next examined SOCS1 mRNA levels in SLE patients and healthy controls and compared them with mRNA levels of the antiviral protein MxA. Fig. 5C, left, shows the results of real time RT-PCR analysis of SOCS1 mRNA, which was markedly decreased in SLE patients (p<0.0001). Although the differences between inactive SLE patients and controls were statistically significant (p = 0.036), active patients showed the greatest differences (p<0.0001). This was also examined after a short stimulation (30 min) with IFNβ to focus mainly on its direct effects upon SOCS1 gene transcription. Fig. 5C shows that IFNβ induces a several fold increase of SOCS1 mRNA levels and, although both initial and final (stimulated) levels were lower in SLE, the increase in SLE and healthy controls was similar (from 3 to 5 fold, Fig. 5A). Although these findings were consistent for most SLE patients, two patients with severely active disease had increased SOCS1 mRNA that further increased upon addition of IFNβ. These patients did not differ clinically or serologically from other similarly active SLE patients. Therefore, we interpret this as a reflection of the heterogeneity in the pathogenesis of SLE. Regardless of this, mRNA levels of SOCS1 did not correlate with disease activity, even when these two patients were excluded from analysis (Not shown). As an important control, mRNA of the anti-viral protein MxA, whose expression is induced by IFN-I, was constitutively increased in SLE patients and further increased in response to IFNβ (Fig. 5C, right) but it did not correlate with disease activity (Fig. S5). The two patients with high SOCS1 mRNA did not differ from other patients in regard to MxA mRNA expression. Thus, SOCS1 expression in response to IFN-I in SLE appears to be dissociated from other IFN-I responsive genes. Taken together, these results indicate that, in SLE, SOCS1 expression is decreased.

Finally, it was important to examine whether decreased SOCS1 was related to the state of Jak1 and/or STAT2 phosphorylation. Figure 5D shows that a large part of SLE patients had an inverse correlation between SOCS1 levels and Jak1 (11 out of 15) and STAT2 (9 out of 15) phosphorylation, suggesting that these patients could fall into two distinguishable groups, one of them related to decreased SOCS1. Nevertheless, Pearson’s correlation analysis of the entire SLE group of patients, failed to reveal a statistically significant association (Fig. S5), in spite of an inverse trend. These findings point again to the heterogeneity in the pathogenesis of SLE and that a better definition of disease subsets could be necessary for this kind of studies to be able to determine the possible role of decreased SOCS1 in constitutive Jak1 and STAT2 phosphorylation in SLE and whether the two possible groups observed by these means behave differently.

### Ligand-dependent Association of SOCS1 with IFNAR2 and Constitutive Association with IFNAR1

**Figure 6:**
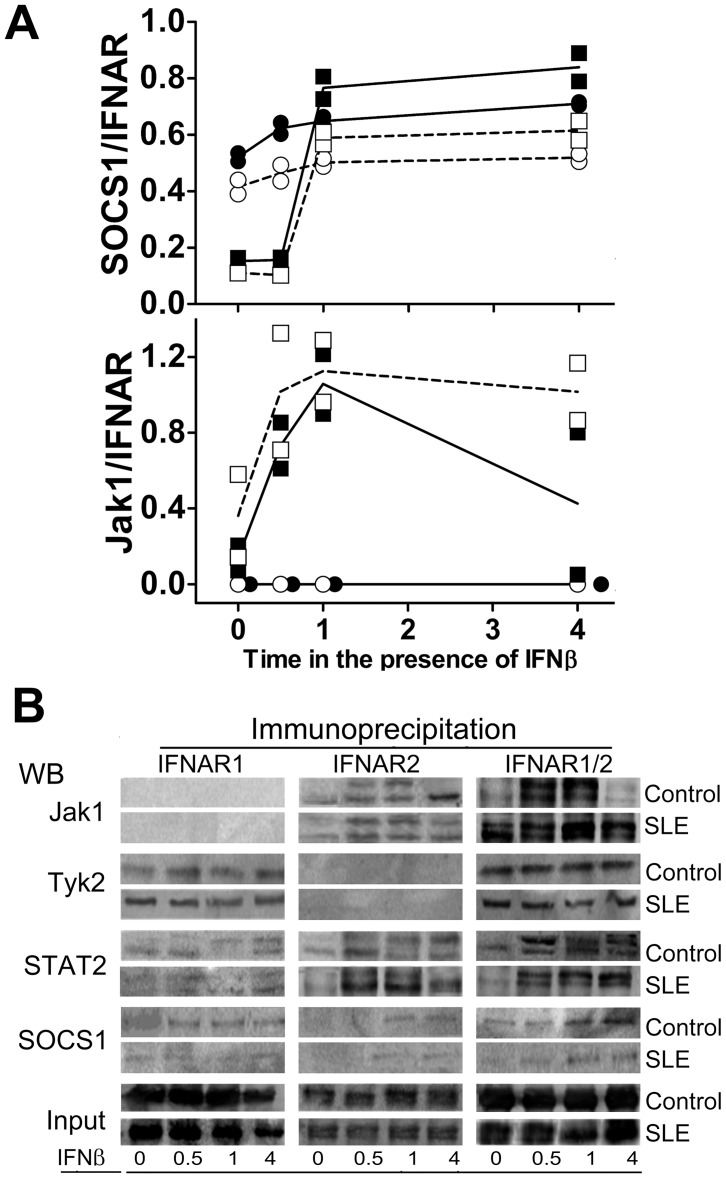
Constitutive association of SOCS1 with IFNAR1 in PBMC. A. Kinetics of SOCS1 (above) and Jak1 (below) association with the individual chains of the IFNAR before (time 0) and after the addition of recombinant IFNβ for the indicated lengths of time (see text). B. One representative Western blot of the indicated proteins obtained from PBMC cell lysates from one healthy control (C) or one SLE patient (P) co-immunoprecipitated with antisera against IFNAR1 and IFNAR2 before (time 0).

Although Jak1 and STAT2 are known to associate to the IFNAR2 chain, it has been suggested that SOCS1 associates only with the IFNAR1 but not with IFNAR2 chain [40]. Therefore, cell lysates from SLE patients or healthy control PBMC were analyzed by coimmunoprecipitation with anti-IFNAR1, IFNAR2, or a mixture of both and blotted with anti-Jak1, Tyk2, STAT2 or SOCS1 antibodies. As expected, Tyk2 was associated with IFNAR1 and Jak1-STAT2 with IFNAR2 (Fig. 6A and 6B). Moreover, as it has been reported [40], in unstimulated PBMC, SOCS1 co-precipitated mainly with IFNAR1.

However, one hour after the addition of IFNβ, SOCS1 association with IFNAR2 increased markedly (about 6 fold), whereas there was only a marginal increase (<1 fold) of IFNAR1-associated SOCS1 (Fig. 6A, 6B). Thus, in the absence of receptor occupation, SOCS1 was associated only with IFNAR1, but ligand binding to IFNAR induces SOCS1 association to IFNAR2. In SLE cells, the amount of SOCS1 bound to IFNAR1 and IFNAR2 was slightly lower than in controls, which is inverse with the amount of Jak1 bound, that was higher in SLE cells and remained bound even 6 hours after the addition of IFNβ, when Jak1 binding to IFNAR in healthy individuals was already subsiding. These results indicate that SOCS1 regulates IFNAR signal transduction through the two receptor chains with different kinetics, where Tyk2-STAT1 is regulated in an early fashion, whereas Jak1-STAT2 regulation is delayed. SOCS1 binding to IFNAR2 has not been previously reported.

## Discussion

These studies were undertaken to examine the status of the IFNAR signaling pathway in SLE. Western blot and immunoprecipitation analysis of SLE PBMC proteins showed constitutive phosphorylation of the IFNAR-associated signaling proteins Jak1 and STAT2, even in inactive patients without increased serum IFNα or IFN-I-like gene-inducing activity. We also found a decrease in SOCS1 expression which did not appear to be related with Jak1/STAT2 phosphorylation. In the absence of exogenous IFN-I, Jak1-STAT2 co-precipitated with IFNAR2, which increased by the addition of IFNβ.

Although SLE susceptibility is genetically heterogeneous [3], [4], [5], [6], [7], most SLE patients share increased expression of IFN-I-induced genes that is more pronounced during disease activity [9], [10], [11], [12], [13], [14], [15], indicating that the different genetic backgrounds leading to SLE converge on the IFN-I signaling pathway. However, serum IFN-I is not always increased in SLE [9], [10], [11], [12], [13], [14], [15] and SLE induced by exogenous IFN-I only occurs occasionally. Thus, it seems reasonable to assume that increased IFN-I only leads to SLE in susceptible individuals. One possibility is that, rather that increased IFN-I production, SLE is associated with an increased responsiveness to IFN-I. The role of IFN-I signaling in SLE is further supported by the finding that absence of IFN-I receptor (IFNAR) in NZB mice prevents SLE-like disease but, interestingly, does not decrease over-expression of IFN-I-induced gene Ifi202 [41]. This does not appear to be universal, however, because in the MRL/*lpr* model of murine SLE, the absence of IFNAR not only fails to prevent the disease but it leads to accelerated autoimmunity, whereas administration of IFN-I increases survival [42], [43].

Although only a few of our active SLE patients had increased levels of plasma or serum IFNα with no correlation with SLEDAI scores, plasmas from active SLE patients induced expression of IFN-dependent genes *in vitro* on control PBMC. Indeed, all active SLE plasmas tested (n = 5) induced mRNA expression of the IFN-dependent genes 2′5OAS, MxA and EIF2α and there was a strong correlation with the SLEDAI scores, particularly for 2′5OAS. This has been previously shown, but it has been attributed to the presence of IFNα, as anti-IFNα can block such activity [38]. We did not detect IFNα in these plasmas despite our ELISA kit is multivalent for IFNα. Thus, it is either a different IFN-I (β, ω), which we consider unlikely because others have failed to find these IFN-Is or, SLE plasma contains, as it has been suggested [38], IFN-I-inducing activity as it could be the case for some RNAs coupled to SLE autoantibodies. In such case, single or double stranded RNA could activate IFN-I transcription via TLR7 or TLR3, respectively TLR [38], [44]. Regardless of that, and more importantly, inactive SLE plasmas lacked such IFN-I-like activity and yet, they had constitutive phosphorylation of Jak1, pointing out again to increased sensitivity to IFN-I as part of the SLE diathesis.

The presence of the IFN signature in SLE has been known for several years, but little has been done to examine the IFNAR signaling pathway and its inhibitors in SLE patients. Dong et al [45] found an increase in STAT1 and its phosphorylated form in kidneys and lymph nodes in MRL/*lpr* mice. Moreover, SOCS1 and SOCS3, which are induced by IFN-I via STAT1 were also increased. However, another study found that inhibition of functional expression of Jak-STAT1 in the kidneys ameliorates the nephropathy in MRL/*lpr* mice [46].

The most striking finding of the current studies was that PBMC from inactive SLE patients with normal serum IFNαα levels, have constitutive phosphorylation of IFNAR signaling molecules, which is not explained by intrinsic hypersensitivity to ligand binding as an exogenous IFNβ induced similar phosphorylation of Jak1 and STAT2 in SLE and control PBMC. As both IFNβ and IFNα bind to IFNAR and do not appear to differ in their biological activities, it seems reasonable to conclude that their activities are interchangeable. This suggests that constitutive activation of IFNAR in SLE is due to an impaired regulation in the absence of ligand.

SOCS1 is the main regulator of signaling through many cytokine receptors, including IFNAR [47]. Thus, we examined whether SOCS1 expression was decreased in SLE as a possible explanation for increased phosphorylation of Jak1 and STAT2 in SLE. We found a decrease in SOCS1 expression, particularly at the mRNA level with decreased *in vitro* response to IFNβ. However, Pearson correlation analysis failed to show association between decreased SOCS1 and pJak1 and/or pSTAT2. A study in Taiwanese SLE patients found an increased expression of CIS, but not SOCS1 or SOCS3 mRNA, whereas a second study in China, found that SLE patients as a whole had increased SOCS1 mRNA expression [48], looking at the latter study in depth it is clear that SOCS1 was increased only in active patients. The differences between these and our results could be explained in part by the genetic heterogeneity of SLE, given the different ethnic groups studied. It is of interest that two of our most active patients had a markedly increased expression of SOCS1 mRNA (but not of the IFN-I induced gene MxA). These two patients had the highest SOCS1 transcription in response to IFNβ, pointing again to the heterogeneity of SLE. However, given that we could not establish a statistical correlation between decreased SOCS1 and receptor phosphorylation, for the moment, the role of SOCS1 on increased IFNAR signaling in SLE should be considered only a possibility.

As previously known, in unstimulated PBMC, SOCS1 co-precipitated only with IFNAR1 [40]. However, upon addition of IFNβ, SOCS1 also associates with IFNAR2 to levels well above those of IFNAR1-associated SOCS1, which only increased slightly. This suggests that initial IFN-I signaling takes place mainly from the IFNAR2 chain until SOCS1 binds to it, when this protein is expected to block IFNAR2 signaling without significantly decreasing IFNAR1 signaling, which would be expected to be initially low, but to remain largely unaffected beyond the expected regulation achieved by constitutive SOCS1 binding.

If decreased SOCS1 turns to be a reproducible feature of SLE, it could explain many of the systemic inflammatory and clinical features of SLE, including weight loss and cachexia, which could be due to impaired regulation of cytokine receptors, including IL-2R [49], IFNγR [50], type III IFN’s [51], IL-12 family receptors [52], [53], as well as toll like [54], and some non-immune receptors such as the leptin receptor [55]. Regardless of its possible meaning, the basis of decreased SOCS1 expression in some SLE patients remains to be elucidated. It could be a SNP in the SOCS1 gene, which seems unlikely, because with such a large proportion of patients having the described phenotype, any GWAS study would have already found an association. Another possibility is that some combinations of gene polymorphisms associated to SLE lead to an impaired regulation of SOCS1 gene expression by a transcriptional factor. A third, more intriguing possibility, is that decreased SOCS1 mRNA is not due to decreased transcription but to increased regulation at the mRNA level, which could be the case if SOCS1 is regulated by microRNA’s. A recent study found that three mouse SLE strains with different genetic backgrounds and pathologic features share a common pattern of altered microRNA expression profiles [56], several of which, including miR-19a, miR-19b, and 155 target SOCS1 mRNA. Moreover, miR-155 is induced by IFN-I [57] resulting in a positive feedback loop that increases IFN-I production.

In conclusion, the finding of constitutive phosphorylation of IFNAR/associated signaling proteins in SLE strongly suggest that overexpression of IFN-I-induced genes in this disease could be explained, at least in part, by an increased sensitivity of IFNAR. The role of decreased SOCS1 on IFNAR increased signaling in some patients remains a possibility that needs to be further examined.

## Materials and Methods

### Patients and Clinical Samples

Peripheral blood was obtained from 43 SLE patients (1992 revised ACR criteria for the classification of SLE [58], Table 1) and age and sex-matched healthy controls recruited from the outpatient clinics at the participating institutions. Disease activity was assessed by the SLE disease activity index (SLEDAI) [59]. SLE patients were classified into inactive (SLEDAI 0), mildly active (SLEDAI 1–4) and severely active (SLEDAI >7). All patients, except two (see methods in Text S1 file), were receiving ≤10 mg prednisone or equivalent/day and no cytotoxic drugs. Patients with kidney or liver failure and those with any other inflammatory condition, including acute or chronic infectious diseases, or any type of cancer were excluded from the study. All individuals studied (patients and controls) were Mexican, consisting of a variable ethnic contribution mainly composed by Amerindians and Spaniards with minor contribution from African and other ethnicities. Thirty one patients were examined for mRNA, whereas a variable number of patients were included in the other experimental categories. Serum IFNα levels were determined by means of a VeriKine Human IFNα ELISA Kit, product No. 41105, Pestka Biomedical Laboratories Inc., Piscataway, New Jersey. This kit detects 14 of the 15 known human IFNα subtypes.

The protocol was approved by the institutional research and ethics committees of the institutions involved (Comisión de Investigación y Comisión de Etica, IMSS; Comité de Investigación en Humanos, INCMNSZ), and all subjects were informed about the protocol contents and gave their written consent to participate in the study. All research was carried out according to the WMA Declaration of Helsinki.

### IFN-I Stimulation *in vitro*

PBMC were isolated on ficoll/sodium ditrizoate gradients (Lymphoprep, Axis-Shield PoC AS, Oslo, Norway) and divided in two aliquots; one for culture in RPMI medium (GIBCO, Invitrogen, Grand Island, NY) supplemented with L-glutamine (2 mM), HEPES (20 mM), penicillin (100 units/ml), streptomycin (100 µg/m), and 10% fetal bovine serum (HyClone, Salt Lake City, Utah) (complete medium, CM) in the presence or absence of 50 U/ml human recombinant IFNβ (Emaxem Probiomed, Mexico City) for different lengths of time. The second fraction was incubated in CM alone or with 50 U/ml of IFNβ for 30 min for RNA extraction.

### Plasma Samples and PBMC Stimulation

Ten ml heparinized blood was centrifuged, and the plasma was recovered and stored at −70°C. PBMC from three healthy controls (ficoll/sodium ditrizoate gradients) were cultured (10^5^/well in 100 µl) in 96-well flat-bottomed plates containing CM, 50 U/mL IFNβ, or healthy control or SLE plasma (50% on CM). After 1 hour of incubation, RNA was extracted from each PBMC fraction (Trizol). 15 ng total RNA was amplified in a 10 µl real-time quantitative RT-PCR reaction using 90 mM sense and antisense primers and 25 µl 2X SYBR green reaction mix with ROX (InVitrogen). Primer sequences were: For EIF2α, 5′ - TTG CTT CAA AAA CAT TCT TAC ATT TT-3′ (forward) and 5′- GGG CAA CAG AGC GAG ACT-3′ (reverse); for 2′5′OAS, 5′- GAG GGG GTG GAG TTC GAT-3′ (forward) and 5′- GGT TAG GTT TAT AGC TGC CAG TCA-3′ (reverse); MxA 5′- GAT GTC CCG GAT CTG ACT CT-3′(forward) and 5′- TGG ATG TAC TTC TTG ATG AGT GTC T-3′ (reverse). Real-time instrument was programmed as follows: 50°C for 3 minutes hold; 95°C for 5 minutes hold; 40 cycles of: 95°C for 15 seconds; 60°C for 30 seconds; 40°C for 1 minute.

### Protein Extraction and Western Blotting

PBMC were lysed in buffer and total cell proteins were quantified as described [60] and run (20 µg) on 12% SDS-PAGE. Gels were transferred onto PVDF membranes (Bio-Rad), blocked and incubated overnight with the different antisera. GAPDH was used as loading control. (See also supporting method files for all experimental details).

### Pull-down Experiments

Cells incubated in medium alone or with 50 U/ml IFNβ for 0, 30 min, 1 h and 4 h were lysed, centrifuged and 50 µg total protein was incubated overnight with 1 mg/ml mouse anti-IFNAR1 and/or rabbit anti-IFNAR2 (either individually or mixed) at 4°C.Lysates were incubated overnight with 100 µl protein G-Sepharose 4B (for IFNAR2) or protein A-Sepharose 4B (for IFNAR1) (Amersham-Pharmacia). Proteins were collected and resuspended in 5x Laemmli buffer. Samples were run on 12% SDS-PAGE, transferred onto PDVF and blotted with antibodies against SOCS1, STAT2, Jak1 and Tyk2 (1 mg/ml). For these experiments the load control (input) was based on the Ig heavy chain of the antibodies used for immunoprecipitation.

### Real Time RT-PCR

Total cell RNA was isolated by means of Trizol reagent (Invitrogen) and reverse transcribed to synthesize single stranded cDNA with the High capacity cDNA reverse transcription kit (Applied Biosystems [ABI], Foster City, CA). Quantitative real time RT-PCR was achieved in the Prism 7900 RT (ABI). Oligonucleotides used were: SOCS1 FAM 5′[GCCAGCGGAACTGCTTTT]3′ sense, and 5′[AGTGCACGCGGATGCT]3′ antisense, MxA FAM (ABI). GAPDH VIC oligonucleotides were used as internal control.

### Statistical Analysis

Statistical analysis was achieved by the Student´s t test for non-paired samples with a 95% confidence limit and significance was set at p<0.05. Simple linear regressions and Pearson’s correlations were achieved to correlate among different parameters with a 95% confidence level.

## Supporting Information

Figure S1 **Similar expression levels of IFNAR1 and IFNAR2 chains in SLE patients and healthy individuals.** Densitrometic values of IFNAR1/GAPDH and IFNAR2/GAPDH western blot analysis of cells lysates obtained from PBMC from SLE patients (squares, n = 15) or healthy controls (circles) before (time 0) and at different times after the addition of 50 U/ml human recombinant IFNβ. Inactive SLE includes patients with SLEDAI 0–4 and active SLE comprises patients with SLEDAI>4.
(TIF)

Figure S2 **Lack of correlation between** c**onstitutive phosphorylation of Jak1 and disease activity in SLE.** Logistic regression analysis of pJak1/GAPDH ratios in SLE patients (Y axes) and disease activity (SLEDAI) indices (X axes) before (time 0) or at the indicated times after the addition of 50 U/ml IFNβ.
(TIF)

Figure S3 **Lack of correlation between** c**onstitutive phosphorylation of STAT2 and disease activity in SLE.** Logistic regression analysis of pSTAT2/GAPDH ratios in SLE patients (Y axes) and disease activity (SLEDAI) indices (X axes) before (time 0) or at the indicated times after the addition of 50 U/ml IFNβ.
(TIF)

Figure S4 **Decreased SOCS1 protein expression in SLE patients does not correlate with disease activity.** PBMC cell lysates from 15 SLE patients were run in PAGE gels, transferred onto PDVF membranes and blotted with anti-SOCS1 antiserum. Data on the Y colum represent the relative SOCS1/GAPDH protein levels. Data from SLE patients were plotted to examine the correlation with disease activity according to the SLEDAI scores and are expressed as arbitrary units. Time 0 represent lysates from cells without the addition of exogenous IFNβ, whereas columns numbered 1, 4 and 6 refer to the time elapsed after the addition of 50 U/ml human recombinant IFNβ.
(TIF)

Figure S5 **Decreased SOCS1 and MxA mRNA expression in SLE patients does not correlate with disease activity.** mRNA samples from 31 SLE patients (left) were examined by real time RT-PCR with human SOCS1 or MxA-specific Taqman probes. Data on the Y column represent the relative SOCS1/GAPDH (top) or MxA/GAPDH (bottom) mRNA levels, both in the absence (left) or in the presence (right) of 50 U/ml human recombinant IFNβ for 30 min. Data were plotted to examine the correlation with disease activity according to the SLEDAI scores and are expressed as arbitrary units. Numbers shown in each graph represent the Pearson’s correlation figures for each type of comparison.
(TIF)

Table S1 Densitometric values of pJak1 in controls and SLE. Data corresponding to graphs shown in figure 1.
(PDF)

Table S2 Densitometric values of pSTAT2 in controls and SLE. Data corresponding to graphs shown in figure 2.
(PDF)

Table S3 Densitometric values of SOCS1 in controls and SLE. Data corresponding to graphs shown in figure 5.
(PDF)

Table S4 Statistics of pJak1 densitometric values in SLE and healthy subjects. Data corresponds to graphs shown in figure 1.
(PDF)

Table S5 Statistics of pSTAT2 densitometric values in SLE and healthy subjects. Data corresponds to graphs shown in figure 2.
(PDF)

Table S6 Statistics of SOCS1 densitometric values in SLE and healthy controls. Data corresponds to graphs shown in figure 5.
(PDF)

Text S1 **Additional methods employed in these studies.**
(DOCX)
