# Supplementary figures and images for: Constitutive Phosphorylation of Interferon Receptor A-Associated Signaling Proteins in Systemic Lupus Erythematosus
Source: PLoS One. 2012 Jul 30;7(7):e41414. doi: 10.1371/journal.pone.0041414 (PMC3408474; doi:10.1371/journal.pone.0041414)

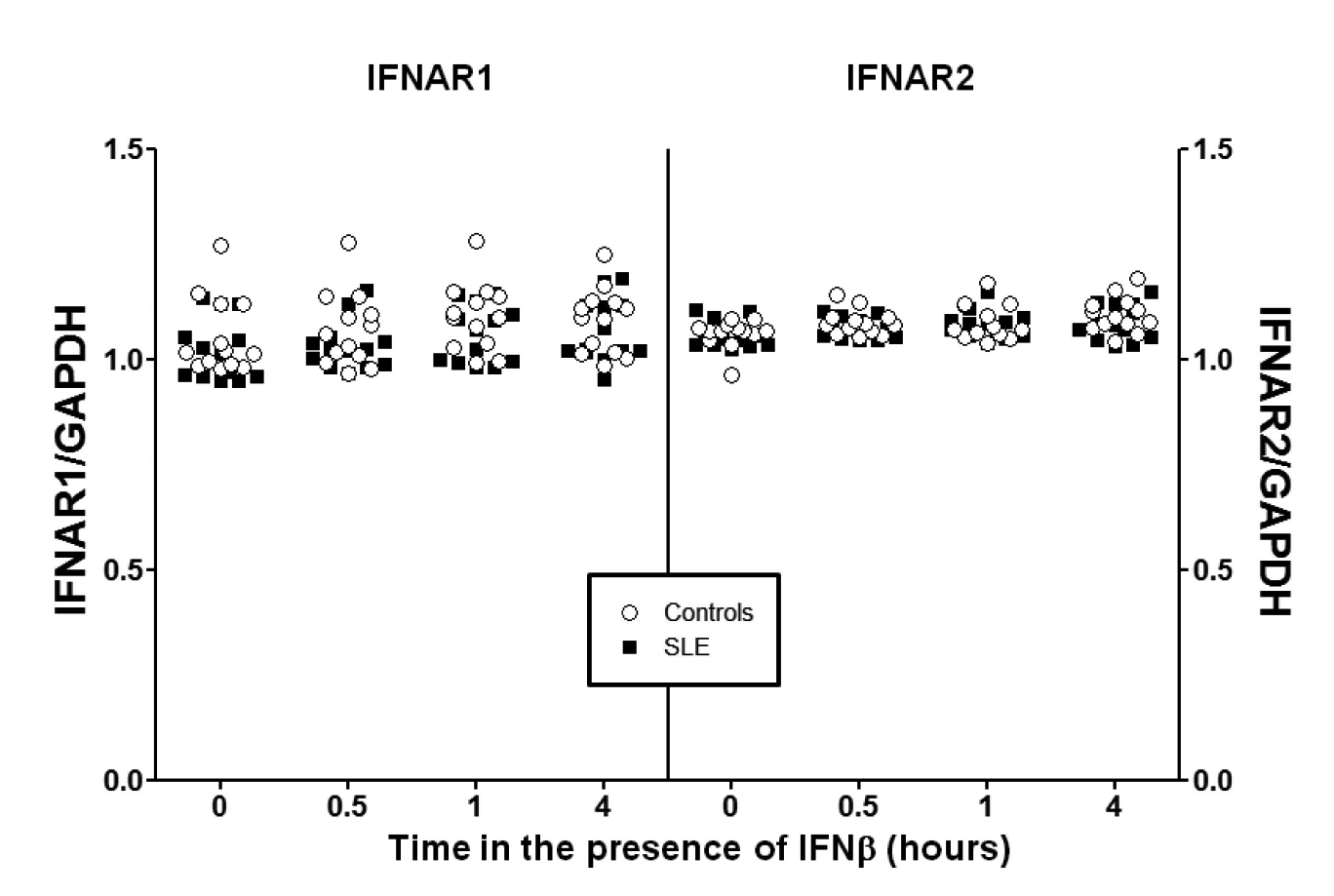

Supplement: Figure S1 — Similar expression levels of IFNAR1 and IFNAR2 chains in SLE patients and healthy individuals. Densitrometic values of IFNAR1/GAPDH and IFNAR2/GAPDH western blot analysis of cells lysates obtained from PBMC from SLE patients (squares, n = 15) or healthy controls (circles) before (time 0) and at different times after the addition of 50 U/ml human recombinant IFNβ. Inactive SLE includes patients with SLEDAI 0–4 and active SLE comprises patients with SLEDAI>4. (TIF) [file pone.0041414.s001.tif]

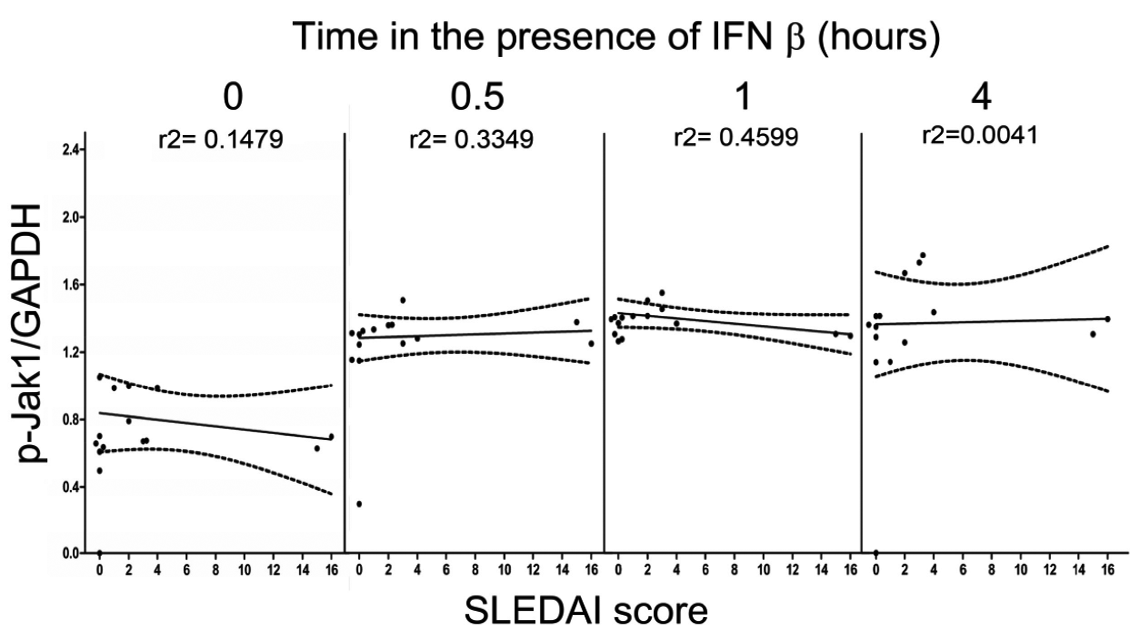

Supplement: Figure S2 — Lack of correlation between constitutive phosphorylation of Jak1 and disease activity in SLE. Logistic regression analysis of pJak1/GAPDH ratios in SLE patients (Y axes) and disease activity (SLEDAI) indices (X axes) before (time 0) or at the indicated times after the addition of 50 U/ml IFNβ. (TIF) [file pone.0041414.s002.tif]

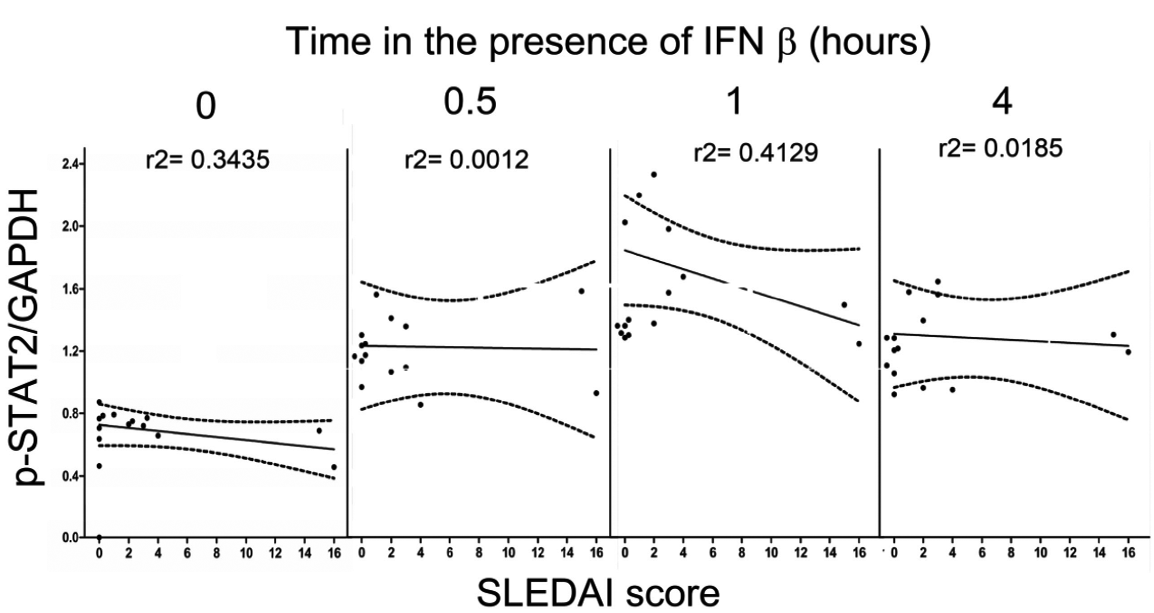

Supplement: Figure S3 — Lack of correlation between constitutive phosphorylation of STAT2 and disease activity in SLE. Logistic regression analysis of pSTAT2/GAPDH ratios in SLE patients (Y axes) and disease activity (SLEDAI) indices (X axes) before (time 0) or at the indicated times after the addition of 50 U/ml IFNβ. (TIF) [file pone.0041414.s003.tif]

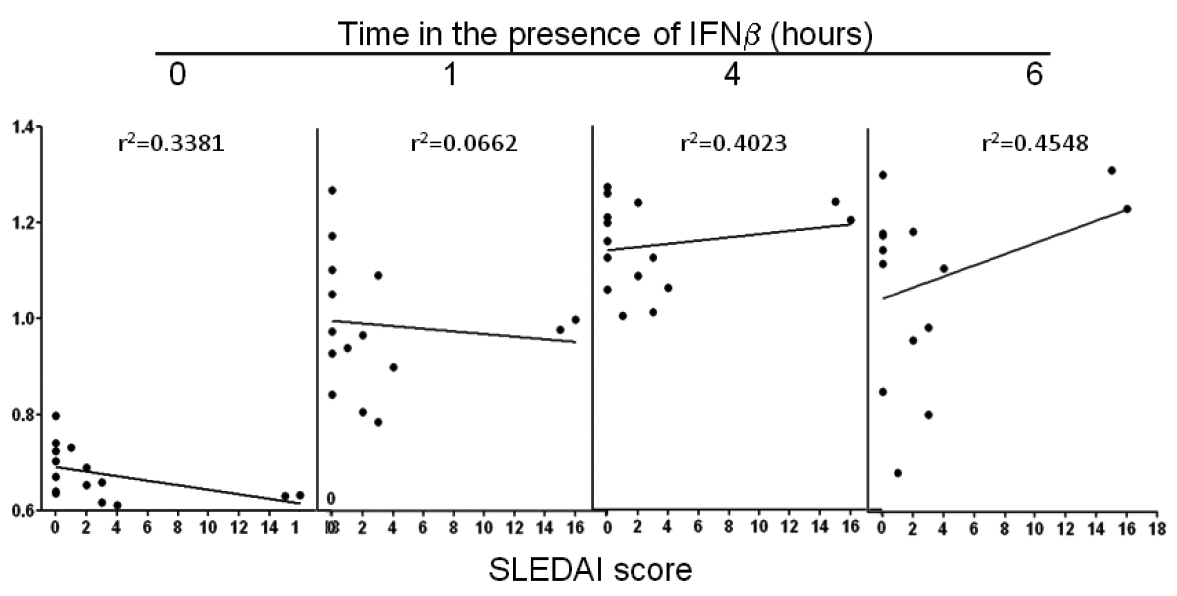

Supplement: Figure S4 — Decreased SOCS1 protein expression in SLE patients does not correlate with disease activity. PBMC cell lysates from 15 SLE patients were run in PAGE gels, transferred onto PDVF membranes and blotted with anti-SOCS1 antiserum. Data on the Y colum represent the relative SOCS1/GAPDH protein levels. Data from SLE patients were plotted to examine the correlation with disease activity according to the SLEDAI scores and are expressed as arbitrary units. Time 0 represent lysates from cells without the addition of exogenous IFNβ, whereas columns numbered 1, 4 and 6 refer to the time elapsed after the addition of 50 U/ml human recombinant IFNβ. (TIF) [file pone.0041414.s004.tif]

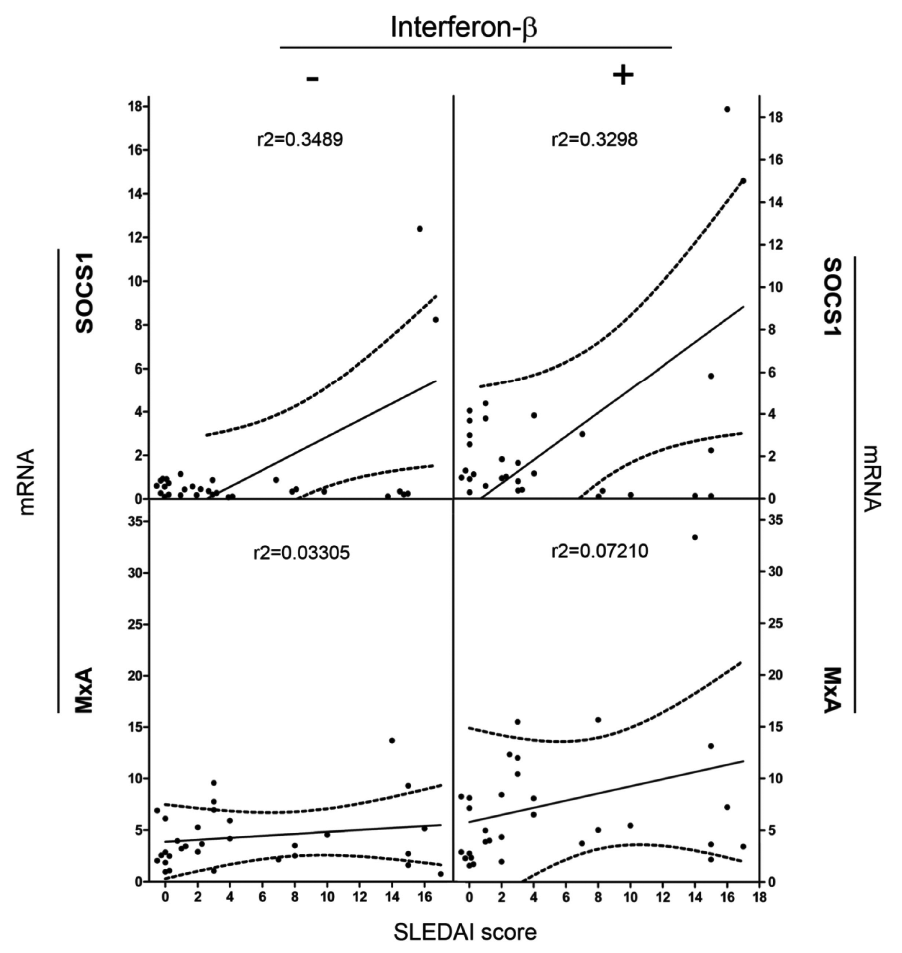

Supplement: Figure S5 — Decreased SOCS1 and MxA mRNA expression in SLE patients does not correlate with disease activity. mRNA samples from 31 SLE patients (left) were examined by real time RT-PCR with human SOCS1 or MxA-specific Taqman probes. Data on the Y column represent the relative SOCS1/GAPDH (top) or MxA/GAPDH (bottom) mRNA levels, both in the absence (left) or in the presence (right) of 50 U/ml human recombinant IFNβ for 30 min. Data were plotted to examine the correlation with disease activity according to the SLEDAI scores and are expressed as arbitrary units. Numbers shown in each graph represent the Pearson’s correlation figures for each type of comparison. (TIF) [file pone.0041414.s005.tif]
