# Supplementary material for: Constitutive Phosphorylation of Interferon Receptor A-Associated Signaling Proteins in Systemic Lupus Erythematosus
Source: PLoS One. 2012 Jul 30;7(7):e41414. doi: 10.1371/journal.pone.0041414 (PMC3408474; doi:10.1371/journal.pone.0041414)
Supplement: Table S1 — Densitometric values of pJak1 in controls and SLE. Data corresponding to graphs shown in figure 1. (PDF) [file pone.0041414.s006.pdf]

Table S1. Densitometric values of pJak1 in controls and SLE

Time with IFNβ (Hours)	Controls	Inactive SLE	Active SLE
0	0.10±0.25	0.59±0.31	0.80±0.16
0.5	1.15±0.1	1.07±0.35	1.29±0.08
1	1.39±0.09	1.35±0.06	1.42±0.09
4	1.04±0.41	1.10±0.49	1.41±0.23
