# Supplementary material for: Constitutive Phosphorylation of Interferon Receptor A-Associated Signaling Proteins in Systemic Lupus Erythematosus
Source: PLoS One. 2012 Jul 30;7(7):e41414. doi: 10.1371/journal.pone.0041414 (PMC3408474; doi:10.1371/journal.pone.0041414)
Supplement: Table S2 — Densitometric values of pSTAT2 in controls and SLE. Data corresponding to graphs shown in figure 2. (PDF) [file pone.0041414.s007.pdf]

Time with IFNβ (Hours)	Table S2. Densitometric values of pSTAT2 in controls and SLE		
	Controls	Inactive SLE	Active SLE
0	0.11±0.15	0.61±0.3	0.70±0.11
0.5	1.13±0.14	1.13±0.1	1.19±0.27
1	1.54±0.39	1.44±0.26	1.74±0.4
4	0.99±0.4	1.16±0.13	1.33±0.27
