# Supplementary material for: Constitutive Phosphorylation of Interferon Receptor A-Associated Signaling Proteins in Systemic Lupus Erythematosus
Source: PLoS One. 2012 Jul 30;7(7):e41414. doi: 10.1371/journal.pone.0041414 (PMC3408474; doi:10.1371/journal.pone.0041414)
Supplement: Table S3 — Densitometric values of SOCS1 in controls and SLE. Data corresponding to graphs shown in figure 5. (PDF) [file pone.0041414.s008.pdf]

Time with IFNβ (Hours)	Table S3. Densitometric values of SOCS1					
	**Controls**		**Inactive SLE**		**Active SLE**	
	**Mean**	**SD**	**Mean**	**SD**	**Mean**	**SD**
**0**	0.74	0.05	0.71	0.04	0.66	0.04
**1**	1.15	0.09	1.05	0.15	0.95	0.11
**4**	1.27	0.08	1.18	0.08	1.12	0.16
**6**	1.32	0.08	1.16	0.13	1.05	0.24
