# Supplementary material for: Constitutive Phosphorylation of Interferon Receptor A-Associated Signaling Proteins in Systemic Lupus Erythematosus
Source: PLoS One. 2012 Jul 30;7(7):e41414. doi: 10.1371/journal.pone.0041414 (PMC3408474; doi:10.1371/journal.pone.0041414)
Supplement: Table S4 — Statistics of pJak1 densitometric values in SLE and healthy subjects. Data corresponds to graphs shown in figure 1. (PDF) [file pone.0041414.s009.pdf]

Table S4. Statistics of pJak1 densitometric values in SLE and healthy subjects				
Group comparison	**IFNβ 50 U/ml (hours)**			
	**0**	**0.5**	**1**	**4**
Controls vs SLE	**p<0.0001**	NS	NS	NS
Controls vs Active SLE	**p<0.0001**	**p=0.0030**	NS	**p=0.0212**
Controls vs Inactive SLE	**p=0.0006**	NS	NS	NS
Active vs Inactive SLE	**p=0.0477**	**p=0.0766**	**p=0.0069**	NS

NS=not significant
