# Supplementary material for: Constitutive Phosphorylation of Interferon Receptor A-Associated Signaling Proteins in Systemic Lupus Erythematosus
Source: PLoS One. 2012 Jul 30;7(7):e41414. doi: 10.1371/journal.pone.0041414 (PMC3408474; doi:10.1371/journal.pone.0041414)
Supplement: Table S5 — Statistics of pSTAT2 densitometric values in SLE and healthy subjects. Data corresponds to graphs shown in figure 2. (PDF) [file pone.0041414.s010.pdf]

Table S5. Statistics of pSTAT2 densitometric values in SLE and healthy subjects.

Group comparison	IFNβ 50 U/ml (hours)			
	0	0.5	1	4
Controls vs SLE	p<0.0001	NS	NS	p=0.0303
Controls vs Active SLE	p<0.0001	NS	NS	p=0.0339
Controls vs Inactive SLE	p<0.0001	NS	NS	NS
Active vs Inactive SLE	NS	NS	p=0.0184	NS

NS=not significant
