# Supplementary material for: Constitutive Phosphorylation of Interferon Receptor A-Associated Signaling Proteins in Systemic Lupus Erythematosus
Source: PLoS One. 2012 Jul 30;7(7):e41414. doi: 10.1371/journal.pone.0041414 (PMC3408474; doi:10.1371/journal.pone.0041414)
Supplement: Table S6 — Statistics of SOCS1 densitometric values in SLE and healthy controls. Data corresponds to graphs shown in figure 5. (PDF) [file pone.0041414.s011.pdf]

Table S6. Statistics of SOCS1 densitometric values in SLE and healthy controls				
Group Comparison	IFNβ 50 U/ml (hours)			
	0	1	4	6
Controls vs SLE	**p=0.0096**	**p=0.0025**	**p=0.0011**	**p<0.0001**
Controls vs Active SLE	**p=0.0007**	**p=0.0002**	**p=0.0058**	**p<0.0001**
Controls vs Inactive SLE	NS	**p=0.0423**	**p=0.0144**	**p=0.0008**
Active vs Inactive SLE	NS	NS	**p=0.0479**	**p=0.0451**

NS=not significant
