# Supplementary material for: Constitutive Phosphorylation of Interferon Receptor A-Associated Signaling Proteins in Systemic Lupus Erythematosus
Source: PLoS One. 2012 Jul 30;7(7):e41414. doi: 10.1371/journal.pone.0041414 (PMC3408474; doi:10.1371/journal.pone.0041414)
Supplement: Text S1 — Additional methods employed in these studies. (DOCX) [file pone.0041414.s012.docx]

**Supporting Text S1 file**

**Additional Methods.**

**Patient data.** One patient was on 50 mg/day prednisone and another with 150 mg/day azathioprine and these two were examined separately from the remaining group.

**Protein Extraction and Western Blotting**. PBMC were lysed in a buffer consisting of TRIS-HCl (Bio-Rad Laboratories, Hercules, CA) 10mM, EDTA (Research Organics Inc, Cleveland, OH) 1mM, NaCl (J.T. Baker, Xalostoc, Mexico) 150mM, 1% Igepal CA-630 (Sigma-Aldrich, Inc, St. Louis, MO), and protease inhibitor cocktail set VII, containing AEBSF, Bestatin, E-64, Pepstatin A and Phosphoramidon (Calbiochem, EMD Biosciences, Inc. La Jolla, CA). Total cell proteins were obtained from the supernatants after centrifugation of cell lysates at 12,000 rpm at 4°C for 15 min. Transferred PVDF membranes (Bio-Rad) were blocked with 5% skim milk/0.05% Tween 20 (Sigma) in PBS pH 7.4 for 30 min at room temperature. Membranes were then incubated overnight with anti-STAT2 (1mg/ml), anti-pSTAT2 (0.5mg/ml), anti-Jak1 (1mg/ml), anti-pJak1 (0.5mg/ml), anti-IFNAR1 (1mg/ml) or anti-IFNAR2 (1mg/ml) or anti-SOCS1 (1mg/ml) at 4°C. Depending on primary antibodies, blots were incubated for 1h with a 1:10,000 dilution of horseradish peroxidase-labeled donkey anti-goat, goat anti-mouse or donkey anti-rabbit IgG, and detected by enhanced chemiluminescence plus substrate system (ECL, Amersham ECL Western Blotting Systems, GE Healthcare, Buckinghamshire, UK) in a Storm gel scanner (GE Healthcare, formerly Molecular Dynamics).

To adjust for possible differences in total protein loaded to each lane, blots were stripped overnight with glycine (Bio-Rad Laboratories, Hercules CA) (0.1M, pH 2.5, 0.5% SDS) at 4°C and re-probed with 1mg/ml goat anti-glyceraldehyde-3-phosphate dehydrogenase (GAPDH) antiserum for 1h at room temperature. Blots were incubated for 1h with 1:10,000 HRP-labeled donkey anti-goat IgG and detected by ECL. Expression levels of all proteins analyzed were normalized to those of GAPDH and examined by densitometry as described in the software manual.

**Densitometry.** Briefly, bands were measured by means of the Kodak 1D Image Analysis Software Windows version 3.5 software using background correction (Eastman Kodak Company, Rochester, NY, USA). The area to be measured was manually adjusted to include the majority of each band without any background. Net intensities in Kodak light units were used to calculate the ratios of problem protein/GAPDH. Values obtained for each band were corrected for the mean of all bands of the same protein divided by its correspondent GAPDH value calculated in the same manner and results represent the data obtained from such calculations.

**Antibodies and reagents.** Antibodies for immunoprecipitations and western blots included: goat anti-SOCS1 (Abcam, Cambridge, MA), rabbit anti-STAT2 (Santa Cruz Biotechnology, Santa Cruz, CA), rabbit anti-phospho-STAT2 (Upstate Biotechnology, Charlottesville, VA), rabbit anti-Jak1 (Santa Cruz), rabbit anti-phospho-Jak1 (Abcam), rabbit anti-Tyk2 (Santa Cruz), mouse anti-IFNAR1 (Santa Cruz) and rabbit anti-IFNAR2 (Santa Cruz). Horseradish peroxidase-labelled donkey anti-goat, goat anti-mouse and donkey anti-rabbit IgG were from Jackson Immunoresearch Laboratories, West. Grove, PA, USA.

**Pull-down experiments.** After incubation with protein G-Sepharose 4B or A-Sepharose

4B, proteins were collected by centrifugation at 12,000 rpm/1min/4°C, washed three times in buffer containing 0.1% Igepal CA-630, and resuspended in 5x Laemmli buffer containing 1% DTT. Samples were heated at 95°C for 5 min and eluted by centrifugation

at 12,000 rpm/5 min. These proteins were run on 12% SDS-PAGE, transferred onto PDVF and blotted with antibodies against SOCS1, STAT2, Jak1 and Tyk2 (1mg/ml).

**Real time RT-PCR**. Total cell RNA was isolated by means of the guanidine isothiocyanate/phenol /chloroform-based extraction (Trizol reagent, InVitrogen). RNA concentration was determined by absorbance at 260nm and its integrity was verified by electrophoresis on non-denaturing 1.2% agarose gels. Total RNA was reverse transcribed and analyzed by quantitative real time RT-PCR Each problem cDNA (SOCS1 and MxA) was amplified by quadruplicate in 384 well plates multiplexed with GAPDH in individual wells. Means of GAPDH cycle thresholds (CT) were subtracted from problem cDNA means for each experimental condition (∆CT). For calculation, control ∆CT was subtracted from patient ∆CT (∆∆CT). For plots, control values were taken as=1, whereas patient gene expression values correspond to the results obtained by the method 2-ΔΔCT (Applied Biosystems User Bulletin No. 2, P/N 4303859).
